# Conceptualizing healthcare professionals’ relational competence in mental healthcare: An integrative review

**DOI:** 10.1016/j.ijnsa.2024.100266

**Published:** 2024-11-08

**Authors:** Lise Sæstad Beyene, Elin Bolle Strand, Aud Ragnhild Misund, Helene Eidsmo Barder, Trine Lise Brente, Hege Therese Størksen

**Affiliations:** a University of Stavanger, Norway; b VID Specialized University, Norway; c Oslo University Hospital, Norway; d Oslo Metropolitan University, Norway

**Keywords:** Integrative review, Mental healthcare, Mental health professionals, Relational competence

## Abstract

**Introduction:**

The relationship between patients and mental health professionals is the cornerstone of all mental health treatment, and good patient-professional relationships are associated with several positive therapeutic outcomes for patients with mental health challenges. Mental health professionals’ relational competence is essential for high-quality services in mental healthcare. There is currently no unified understanding of the concept of relational competence. This study seeks to address this gap by exploring how relational competence is conceptualized within the context of mental healthcare. The research question was: How is relational competence described in the research literature within the context of mental healthcare?

**Methods:**

An integrative review was conducted with systematic searches in the databases PsycInfo, Ovid Medline, Embase, CINAHL, ERIC, Academic Search Elite, IDUN, and Svemed+, spanning from January 2012 to October 2023, as well as hand searches in the reference lists of the included studies. A thematic synthesis was carried out based on the results in the included studies.

**Results:**

Out of 2970 scientific studies screened, 30 were included, employing a variety of research methodologies to explore relational competence within mental healthcare. Four themes were found to describe relational competence in mental healthcare: having the ability to self-reflect and self-regulate, having a genuine interest in understanding the patient, engaging in reciprocal interaction with the patient, and meeting the patient so that they feel acknowledged. Each theme describes a central and important part of relational competence, but fully developed relational competence must be understood as a whole in which all the themes are present.

**Conclusion:**

Relational competence in mental healthcare incorporates all the identified components. Each theme complements the others and contributes to the construction of a strong therapeutic relationship between patients and mental health professionals. To provide the best possible care for mentally ill patients, healthcare professionals must embrace and integrate these elements into their practice.

**What is already known**

• The relationship between patients and mental health professionals is the cornerstone of all mental care treatment, and good patient-professional relationships are associated with several positive therapeutic outcomes for patients with mental health challenges.
• Mental health professionals’ relational competence is essential for high-quality services in mental healthcare.
• Various aspects of relational competence are highlighted in research. Relational competence is operationalized through mental health professionals’ individual focus and understanding.

Alt-text: Unlabelled box

**What this paper adds**

• This paper summarizes four key themes representing relational competence in mental healthcare: self-reflection and self-regulation, genuine interest in understanding the patient, reciprocal interaction with the patient, and ensuring patients feel acknowledged.
• It emphasizes the interconnectedness and interdependence of these themes, highlighting the holistic nature of relational competence.

Alt-text: Unlabelled box

## 1 Introduction

Mental healthcare is a dynamic context where people with emotional challenges, suffering from conditions such as depression, anxiety, schizophrenia, bipolar disorder, and other mental illnesses, receive treatment and care from mental health professionals. Patients and situations within this realm are often inconsistent and changeable, leading to demanding scenarios for mental health professionals, who must be capable of meeting each patient and situation with empathic support and safeguarding patients’ autonomy and human rights. Frontline workers in mental healthcare predominantly include registered nurses, social educators, social workers, and auxiliary nurses. In this context, caring is a therapeutic, interpersonal process (Peplau, 1997), achievable only through the establishment of a real human-to-human relationship (Travelbee, 1971). Patients admitted to mental health wards benefit from nurse-patient therapeutic engagement (McAllister et al., 2019), with the relationship between patient and nurse serving as the cornerstone of all psychological treatment and being associated with several positive therapeutic outcomes (Strandås and Bondas, 2018; Eton et al., 2017). Studies indicate that 30 % of patients’ recovery is contingent upon mental health professionals’ capacity to establish therapeutic relationships that serve as a foundation for health promotion processes (Lambert and Barley, 2001), underscoring the pivotal role of relational competence for high-quality services in mental healthcare.

However, despite the recognized significance of relational competence, there is no unified understanding of the concept. Various aspects of relational competence are highlighted in research. The nurse Joyce Travelbee (Travelbee, 1971) was one of the first to describe in more detail which relational elements are necessary for successful therapy. Travelbee's grand theory of human-to-human relationships emphasizes the therapeutic use of oneself, which she describes as entailing a conscious use of one's own personality and knowledge to contribute to therapeutic change in the patient. In developing therapeutic relationships, healthcare professionals must have the ability to take an interest in the patient as a person, to put themselves in the other's position, show emotions and be a fellow human being and show sympathy, and have a desire to help, as well as having the practical and theoretical knowledge to meet the patient's needs (Travelbee, 1971).

The psychologist Carl Rogers (Rogers, 1977) was also one of the pioneers in emphasizing the meaning of professional relationships. He presented the therapist's ability to be congruent in the relationship, unconditional positive acceptance, and accurate empathic understanding of the patient's experiences as particularly significant in the therapist's way of relating.

Empathy, genuineness and warmth are described as important for progression across various treatment approaches (Norcross and Wampold, 2011; Watson et al., 2014; Elliott et al., 2018) and are more important to therapeutic outcomes than the specific intervention applied (Bohart et al., 2002). Moreover, the therapist's empathy may increase patients’ empathy towards themselves or help them recognize and regulate their emotions, as well as contribute to a safer attachment in the patient (Watson et al., 2014). Empathy is central to the development of therapeutic alliance (Castonguay and Beutler, 2006), which is also an important term in the therapeutic context (Horvath et al., 2011; Ryum and Stiles, 2005).

The therapeutic alliance, which has been extensively explored in psychotherapy research (Flückiger et al., 2018; Wampold, 2015; Wiseman and Tishby, 2014), refers to cooperation on goals and targets for treatment and the emotional bond between therapist and patient (Del Re et al., 2021). It contains several elements and requires that the therapist be flexible, honest and respectful, trustworthy, confident, interested and alert, as well as friendly, warm and open (Ackerman and Hilsenroth, 2003).

Furthermore, emotional intelligence emerges as an important part of relational competence (Smith et al., 2009; Hen and Goroshit, 2011). To develop therapeutic relationships, one must be able to understand and manage both one's own and the patient's feelings. Emotional intelligence is the ability to perceive, assess, express and regulate emotions in oneself and others (Salovey and Mayer, 1990), which will impact the quality of patient care and outcomes (Raghubir, 2018). Good mental capacity or self-reflection is essential to distinguish between emotions and differentiate one's own feelings from those of others. Awareness of one's emotional attitude and emotional regulation strategies also requires some capacity for mentalization and self-reflection (Hen and Goroshit, 2011).

An integrative review conceptualizing nurse-patient therapeutic engagement in acute inpatient settings identified five principles, including “understanding the person and their experiences”, “facilitating growth”, “therapeutic use of self”, “choosing the right approach” and “authoritative vs. emotional containment” (McAllister et al., 2019). This underscores the active interaction between mental health professional and patient, in which relational competence serves as a prerequisite for establishing therapeutic engagement and health-promoting relations in mental healthcare.

In education and training for mental health professionals, relational competence is addressed by imparting knowledge about and developing students’ relational skills. Relational competence is a complex concept; thus, it is important to understand the elements comprising relation competence to better organize education and guidance, adapt therapeutic challenges in mental health practice, and evaluate the quality of care. Despite its recognized importance, relational competence is not clearly defined in the literature. Therefore, this study seeks to address this gap by exploring how relational competence is conceptualized within the context of mental healthcare, grounded in the human science perspective (Eriksson, 2002). The research question guiding this review was: How is relational competence described in the research literature within the context of mental healthcare?

## 2 Methods

We conducted an integrative review, which had the purpose of summarizing past empirical and theoretical literature to provide a more comprehensive understanding of relational competence in the context of mental healthcare. The integrative review method is an approach that allows for the inclusion of diverse methodologies (Whittemore and Knafl, 2005). Due to this, the appraisal and inclusion of evidence in an integrative review is not limited by the methodological quality of the evidence (Whittemore and Knafl, 2005), and we did not aim to select studies according to their methodological or evidence quality. We based the structure of this integrative review on Whittemore and Knafl's (Whittemore and Knafl, 2005) methodological framework for integrative reviews. In the first step, we identified the problem by formulating research questions related to the concept of relational competence among healthcare professionals in mental healthcare settings. In the second step, we evaluated the data by reviewing the extracted information for relevance and quality, and we excluded articles that did not meet our inclusion criteria. In the third step, we conducted a thematic analysis to code and organize the extracted data into meaningful categories. Finally, in the fourth step, we presented the results of our analysis in a narrative format, highlighting the key findings and their implications for clinical practice and future research. This framework contributes to enhancing rigor and transparency of integrative reviews and helps to enhance the reliability of the findings. This review was registered in Prospero in May 2022 (CRD42022323986).

### 2.1 Literature search stage

A comprehensive literature search was conducted in the following scientific databases: 1) PsycInfo, 2) Ovid Medline, 3) Embase, 4) CINAHL, 5) ERIC, 6) Academic search elite, 7) IDUN and 8) Svemed+. The search strategy was developed in collaboration with a research librarian who performed the database searches on 9 June 2022. In addition to the initial search, an update search was performed on 23 October 2023, to ensure the inclusion of the most recent studies. The PsycINFO search strategy (for the complete search strategy, see Appendix 1) was tailored to other databases using appropriate subject headings and terms; hence, search strings varied slightly depending on the indexing and descriptors used in the various databases. The search strategies consisted of a combination of index and free text terms describing 1) relational competence and 2) mental health settings. After duplicate checks, the search results were imported into Rayaan (www.rayyan.ai), a systematic review management tool, for the study selection process.

#### 2.1.1 Study selection

A two-stage screening process was carried out whereby all references were, first, considered for inclusion based on their title and abstract by two members of the research team independently (LSB and HEB, EBS and TLB, ARM and HTS). Second, all included references were retrieved in full-text and screened independently by two raters (LSB and EBS, HEB and ARM, TLB and HTS). Any disagreements were resolved by discussion or a third rater (LSB or HTS). Eligible articles had to include 1) descriptions and/or definitions of relational competence, 2) healthcare professionals and 3) a mental healthcare setting, specifically targeting the adult population. We included all empirical, theoretical, and methodological studies and systematic reviews fulfilling the inclusion criteria and excluded references such as editorials, study protocols and viewpoints. We also excluded studies that only included students as participants as our focus was on the relational competence of professional healthcare providers. Additionally, we excluded studies that described relational competence in settings not related purely to mental health, such as social work and somatic hospital settings. Furthermore, digital settings were also excluded as we aimed to focus on relational competence in a physical setting. Our review focused on articles published in peer-reviewed journals in English. Studies published within 10 years prior to the first search date were included. The decision to exclude studies published before 2012 was based on several factors, including relevance to current practices, manageability of the review and specific inclusion criteria. After the initial screening process, we also hand-searched reference lists in identified articles.

#### 2.1.2 Data evaluation stage

A tailored approach was taken to assess the quality of the included articles based on their respective study designs. Specifically, the Critical Appraisal Skills Program (CASP) was used to evaluate the quality of qualitative studies (LSB and TLB) (Long et al., 2020), the Mixed Methods Appraisal Tool was used to evaluate the quality of mixed methods studies (EBS and ARM) (Hong et al., 2018) and the Joanna Briggs Institute (JBI) critical appraisal checklist was utilized for cross-sectional studies and literature reviews (HEB and HTS) (Institute, 2017). The National Institutes of Health's Quality Assessment Tool was applied for the assessment of pre-post studies (National Heart L, and Blood Institute 2013). Integrative reviews consider a wide range of evidence; notably, no article was excluded based on the quality assessment (Whittemore and Knafl, 2005).

#### 2.1.3 Data extraction

The data extraction process was rigorous, with all authors (LSB, HEB, EBS, TLB, ARM and HTS) involved in the initial extraction process. To ensure data accuracy and consistency, a Microsoft Word table was developed, and extracted data were systematically reviewed by LSB to ensure data quality. The extracted data included the first author, title, year of publication, country, description or definition of relational competence, type of healthcare professionals, and mental healthcare setting (Table 1).

**Table 1:** Description of included studies.

Author, year	Country	Aim, objective/ purpose	Sample	Context	Method	Results	Quality assessment score
Andvig, E; Biong, S. (2014)	Norway	To describe and explore what health professionals focused on in recovery-oriented conversations with patients in a Norwegian mental healthcare centre	15 milieu therapists	A milieu therapeutic setting	Action research project with qualitative and explorative design	The findings highlighted the prerequisites for conversation, the content of conversation and different views on the topics of conversation	High (CASP)
Basogul, C; Arabaci, L; Buyukbayram, A; Aktas, Y; Uzunoglu, G. (2019)	Turkey	To examine the relationship between the emotional intelligence and sociotropic-autonomic personality characteristics of nurses working in psychiatry clinics and their exposure to violence	103 nurses	Mental health community hospital	Cross-sectional, correlational design	High emotional intelligence levels and autonomous personality characteristics decrease the rate of exposure to violence. However, sociotropic personality characteristics increase the rate of exposure to psychological violence.	Medium (JBI)
Battles, M.B; Berman, J.S. (2012)	USA	To examine two conversational acknowledgers in psychotherapy and evaluate the effects of therapists varying levels of two specific actions (short utterances and nodding), on perceptions of the therapist	320 undergraduate students	Therapy interactions between a therapist and a pseudoclient	Quantitative analysis of a series of brief psychotherapy videos	When therapists used high levels of both verbal acknowledgers and nodding, or when they refrained from using both types, therapist empathy and therapeutic alliance were perceived as greater than when therapists engaged in one type of acknowledger but not the other	Low (JBI)
Bhola, P; Kumaria, S; Orlinsky, D.E. (2012)	India	To understand the experiences and contexts of psychotherapists’ practice in India	250 psycho-therapists	Psychotherapists’ practices	Consensual qualitative research method	Themes related to therapists’ perceived strengths: therapeutic relationship skills, therapeutic professional expertise, and therapist personal qualities and experiences. Four themes accounted for therapists’ views of their limitations: inadequate therapeutic competence, professional stress and burnout, inadequate professional knowledge and experience and therapist personal qualities and difficult experiences	Low (CASP)
Brekke, E; Lien, L; Biong, S. (2017)	Norway	To explore and describe behaviour and attributes of professional helpers that support recovery, as experienced by persons with co-occurring disorders	8 persons with co-occurring disorders	Community mental health and addiction services in a local authority area	In-depth individual interviews analysed using systematic text condensation	The analysis yielded four categories of recovery-supporting behaviour and attributes of professional helpers and the ability to build trust cuts across all of them: Building trust through(a) hopefulness and loving concern, (b) commitment, (c) direct honesty and expectation and (d) action and courage	High (CASP)
Brown, B; Crawford, P; Gilbert, P; Gilbert, J; Gale, C. (2014)	UK	Examine practitioners’ accounts of compassion in their daily work and explore how they formulated, interpreted and deployed the concept and its concomitant practices in the context of their working lives	20 mental health practitioners	An in-patient mental health facility	Qualitative interviews analysed by means of constructionist discourse analysis	The participants drew on two key repertoires. The practical compassion repertoire, focused on the practice of compassion through support, practice and meaning, whereas the organisational repertoire, focused on a variety of contextual factors that reduced the availability of compassionate care for patients	Medium (CASP)
Carmona-Navarro, M; Pichardo-Martinez, M. (2012)	Spain	To assess attitudes and the influence of emotional intelligence	81 nursing professionals	Emergency and mental health services	Cross-sectional survey	The results show a general adverse attitude towards suicidal behavior. The moral dimension of suicide makes the differences between mental health and emergency professionals. The formation and development of emotional skills are essential for care delivery to patients with suicidal behavior	Medium (JBI)
Evatt, M; Scanlan, J.N. (2022)	Australia	To explore mental health occupational therapists’ understanding of the therapeutic relationship.	14 occupation therapists	Mental health care	Thematic analysis	Three themes were identified: (i) the importance of positive engagement, (ii) specific strategies and techniques used to initiate this relationship and (iii) maintaining boundaries. The study further indicated that the development of therapeutic rapport takes time and opportunities for therapists to engage with service users in seemingly "non-directed" activities should be valued and retained.	Moderate (CASP)
Gerace, A; Oster, C; O'Kane, D; Hayman, C. L; Muir-Cochrane, E. (2018)	Australia	To explore how empathic processes operate when there is conflict between mental health nurses and consumers, and how empathic understanding can be accomplished to facilitate conflict resolution and positive consumer outcomes	13 mental health nurses and 7 consumers	Psychiatric inpatient units	Semistructured interviews analysed by means of thematic analysis	The central theme identified was ‘my role as a nurse–the role of my nurse’. Within this theme, nurses focussed on how their role in managing risk and safety determined empathy experienced towards consumers; consumers saw the importance of nurse empathy both in conflict situations and for their general hospitalization experience	Medium (CASP)
Gunasekara, I; Patterson, S; Scott, J. G. (2017)	Australia	To explore service users’ experiences and expectations of psychiatrists and consultations, engaging psychiatrists throughout the process	22 inpatient service users	A public mental health service providing services to 330,000 residents	An iterative qualitative study with individual interviews and analysed based on a framework method	As ‘masters of their craft’, excellent mental health doctors engage authentically with service users as people (not diagnoses). They listen, validate experiences, and empathise affectively and cognitively. They demonstrate phronesis, applying clinical knowledge compassionately	Medium (CASP)
Karman, P; Kool, N; Gamel, C; van Meijel, B. (2015)	Netherlands	To investigate professional behavior of mental health nurses with positively changed attitudes after following a training program	11 mental health nurses	3 mental health organizations; Inpatient and outpatient facilities with various incidence of self-harm	Grounded theory, semi-structured interviews	Participants reported using less restrictive interventions, being more patient-oriented, and choosing a more empathic and exploratory approach after the training. A work environment conductive to making autonomous professional decisions with supportive colleagues enabled these changes	High (CASP)
Lamke, D; Catlin, A; Mason-Chadd, M. (2014)	USA	To evaluate the effect of training nurses in Jin Shin Jyutsu® self-care methods and to correlate the training with measurement of the nurses’ personal and organizational stress and their perceptions of their caring efficacy for patients	20 nurses	Health care clinics and hospital	A quasi-experimental, pretest, posttest, and 30-to 40-day posttest design	Participants reported significant increases in positive outlook, gratitude, motivation, calmness, and communication effectiveness and significant decreases in anger, resentfulness, depression, stress symptoms, time pressure, and morale issues after the training. There was also a statistically significant increases in nurses’ caring efficacy in areas of serenity in giving care, tuning in to patients, relating to patients, providing culturally congruent care, individualization of patient care, ability to decrease stressful situations, planning for multiple needs, and creativity in care	Low (NIHQ)
Li, X; Kivlighan, D. M; Hill, C. E. (2020)	USA	To examine the effects of therapist interpersonal responsiveness on client-rated working alliance in their first psychotherapy session	111 clients and 38 therapists	Adult community clients in a university clinic	The ordinary differential equations (ODE) model and multilevel data disaggregation	Client-rated working alliance was (a) highest for therapists who generally increased their level of dominance/ submission when their client was more dominant/submissive in the previous turn, and (b) lowest for therapists who generally increased their level of dominance/submission when their client was more submissive/dominant in the previous turn	Medium (JBI)
Liu, S-J; Wang, Q-N; She, J; Zhang, Y-H; Xu, H. (2023)	China	To assess the current EI status among psychiatric nurses and to explore the connections between demographic variables, job stress and EI	1083 psychiatric nurses	Pychiatric nurses working in the clinical frontline.	Cross-sectional study	The findings revealed a noteworthy negative correlation between nurse job stressors and emotional intelligence. Socio-demographic factors and job stressors of certain nurses were able to predict emotional intelligence and its dimensions among psychiatric nurses, with percentages of 44.50 %, 40.10 %, 36.40 %, 36.60 % and 34.60 %	High (JBI)
Ljungberg, A; Denhov, A; Topor, A. (2015)	Sweden	To synthesize the available qualitative research to acquire a deepened understanding of what helpful relationships with professionals consists of, from the perspective of persons with severe mental illness	21 articles	Helpful relationships with mental health professionals	Meta-ethnography	The results show that it can be helpful to individuals to have a relationship where they get to spend time with professionals that are known and trusted, who give them access to appreciated support, collaborative work and valued interpersonal processes, which is allowed to go beyond the boundaries of the professional relationship	High (JBI)
Ljungberg, A; Denhov, A; Topor, A. (2015b)	Sweden	To review the available qualitative research providing knowledge of non-helpful relationships from the perspective of persons with severe mental illness	17 articles	Non-helpful relationships with mental health professionals	Literature review	The main themes were ‘‘non-helpful professionals’’, ‘‘organization versus relation’’ and ‘‘the consequences of non-helpful relationships with professionals’’. Examples of professionals described as non-helpful were pessimistic and uncaring professionals who were paternalistic and disrespectful. Discontinuity, insufficient time and coercion were some of the contextual factors described as non-helpful.	High (JBI)
Loughland, C; Kelly, B; Ditton-Phare, P; Sandhu, H; Vamos, M; Outram, S; Levin, T. (2015)	Australia	To evaluate a pilot communication skills education program for psychiatry trainees, focusing on discussing schizophrenia diagnosis and prognosis	38 junior medical officers	Psychiatric trainees in a formal education program	Pre-post study design	The results showed significant improvements in confidence reported post training for discussing schizophrenia prognosis, including an increased capacity to critically evaluate their own communication skills	Low (NIHQ)
Moltu, C; Binder, P-E. (2014)	Norway	How skilled therapists from various affiliations experience their own contribution in specific therapies, and how they integrate therapeutic techniques	12 experienced therapists	Psychotherapy from different therapy modalities delivered in naturalistic settings	An explorative and reflexive systematic qualitative approach	Results report the overarching theme of maintaining double awareness to provide a relational space for growth, achieved through three concrete themes: (1) tailoring the therapeutic frame to relational struggles, (2) using embodied empathy, and (3) creating meaning from the perspective of a theoretical model	High (CASP)
Moreno-Poyato, A. R; El Abidi, K; Rodriguez-Nogueira, O; Lluch-Canut, T; Puig-Llobet, M. (2021)	Spain	To explore the perspective of people who had experienced treatment as patients at acute mental health units, regarding an intervention model to improve therapeutic relationships in the units, which had been previously designed by the nurses	11 patients	Mental health units	Six focus groups analysed by the use of content analysis method	The results were classified into three themes: (a) the meaning of a space to enable the establishment of a therapeutic relationship, (b) the procedures to implement the space, and (c) the difficulties to overcome to establish the space	High (CASP)
Moreno‐Poyato, A. R; Rodríguez‐Nogueira, Ó. (2021)	Spain	To examine whether the dimensions of empathy influence the nurse–patient therapeutic relationship within mental health units	198 nurses	Mental health units	Cross-sectional study design	Nurses established a greater therapeutic alliance with patients when they were able to adopt their patient's perspective and experience concern	Medium (JBI)
Murphy, D; Elliott, R; Carrick, L. (2019)	UK	To identify facilitative therapeutic principles in person-centred and emotion-focused therapy for working with traumatised clients in the early stages of therapy	4 cases	Therapy sessions	Qualitative, bottom-up inductive process analysis	Four trauma-focused therapist principles were identified: (a) support early relationship building, (b) facilitate identification/recognition of past events as client trauma experience, (c) facilitate work on traumatic sources of current experiential/interpersonal difficulties, and (d) offer self-agency focused empathy	High (CASP)
Olson, D. A; Westra, H. A.; Shukla, S; Di Bartolomeo, A. A (2023)	USA	To analyse therapy disagreement episodes with precise coding measures that capture moment-to-moment sequences of therapist and client utterances	60 disagreement episodes	Therapy sessions	Microlevel, moment-to-moment coding	While appropriate responsivity did not predict outcome, responsivity errors significantly predicted poorer outcome at one year post-treatment.	Moderate (JBI)
Omylinska‐Thurston, J; McMeekin, A; Walton, P; Proctor, G. (2019)	UK	To explore clients` perceptions of unhelpful factors in CBT in IAPT serving a deprived area	9 clients	Therapeutic settings	Thematic content analysis	Results were categorised into six areas: (1) Difficulties with CBT itself, (2) Negative perception of therapists, (3) Clients' unhelpful internal patterns, (4) Physical health, mental health and psychosocial barriers, (5) Unhelpful IAPT processes, (6) Consequences of unhelpful treatment	High (CASP)
Russell, K. A; Swift, J. K; Penix, E. A; Whipple, J. L (2022)	USA	To examined participants’ preferences for personality characteristics of an ideal therapist.	335 psychotherapy clients	Therapeutic settings	Cross-sectional study	Results suggest that clients desire a therapist who is high in emotional stability and conscientiousness; however, participants also highly preferred their ideal therapists display agreeableness. Openness and extraversion were less important to clients	Moderate (JBI)
Romeu‐Labayen, M; Tort‐Nasarre, G; Rigol Cuadra, M.A; Giralt Palou, R; Galbany‐Estragués, P. (2022)	Spain	To describe how people diagnosed with borderline personality disorder (BPD) who have experienced an improvement, perceive the role of the attitudes of mental health nurses in building a positive therapeutic relationship	12 women diagnosed with BPD	Community Mental health service/treatment	Thematic content analysis	Five attitudes were identified of mental health nurses as contributing to a positive therapeutic relationship: confidence in their ability to recover, non-judgement, humour, availability and humanity.	Medium (CASP)
Sovold,L.E; Solbakken, O.A. (2022)	Norway	To investigate and synthesize the different aspects of users’ experiences with therapeutic health interventions across user groups and contexts	30 articles	Psychotherapeutic interventions in clinical settings	Scoping review	Authors identified five main categories or dimensions relevant to the user experience with therapeutic interventions: 1) Perception of self as patient/user, 2) Perception of the therapist/intervention provider, 3) Perception of the therapeutic relationship/alliance, 4) Perception of the intervention/ modality and 5) Perception of contextual factors.	High (CASP)
Tane, E; Fletcher, I; Bensa, S. (2022)	UK	To explore staff's understanding and conceptualisation of the development, loss and restoration of compassion	11 participants from a variety of professional backgrounds	Acute inpatient environments	Grounded theory	A conceptual model of the facilitators and inhibitors of compassionate care was developed, based on five categories that emerged from the data: A compassionate stance; the challenges of acute wards; feeling under threat; restoring compassion; and a compassionate organisation.	High (CASP)
Vandewalle, J; Deproost, E; Goossens, P; Verfaillie, J; Debyser, B; Beeckman, D; Van Hecke, A; Verhaeghe, S. (2020)	Belgium	To enhance the conceptual understanding of the working alliance in the context of nursing care for people experiencing suicidal ideation	28 nurses	Wards in psychiatric hospitals	The Qualitative Analysis Guide of Leuven	Authors identified a core variable and three clusters: investing in the foundations of the working alliance, nourishing the clinical dimension of the working alliance and realizing an impact with the working alliance	Medium (CASP)
Zugai, J. S; Stein‐Parbury, J; Roche, M. (2018)	Australia	To explore the nature of the inpatient therapeutic alliance between nurses and consumers with Anorexia Nevrosa (AN)	95 consumers and 85 nurses	Six wards with specialised programme for treatment of AN in five hospitals	A sequential mixed- methods approach	In a therapeutic alliance, nurses cared for consumers with interpersonal finesse, whilst maintaining clear distinction between the consumer as an individual and AN as an illness. Nurses also developed a therapeutic alliance by occupying their position of power with consistent yet individualised expectations and by maintaining appropriate professional boundaries	High (MMAT)
Zugai, J. S; Stein-Parbury, J; Roche, M. (2015)	Australia	To establish a contemporary interpretation of the concept therapeutic alliance for mental health nursing	52 articles	Therapeutic alliance and/or interaction in the context of nursing	An evolutionary concept analysis	Therapeutic alliance is characterised by mutual partnerships between nurses and consumers and is dependent on a humanistic healthcare culture	Medium (CASP)

#### 2.1.4 Data synthesis

After the initial data extraction, a thorough thematic synthesis was conducted using NVivo, a well-established qualitative data analysis software program, to facilitate data management and analysis. The software was used to code the qualitative extracted data into codes and themes, allowing for a more in-depth and nuanced analysis of the data. The first author (LSB) conducted the coding of the qualitative extracted data and systematized the codes into themes. The quantitative extracted data was then coded and systematized into the existing themes by the second author (EBS). The themes were then validated by all authors to examine whether the themes fit in a coherent pattern and whether they reflected the codes and data extracts. The analysis involved a process of constant comparison, whereby emerging themes were compared against the original data to ensure the integrity and accuracy of the findings (Thomas and Harden, 2008). The use of NVivo allowed for a more comprehensive and rigorous analysis of the data, enabling the research team to draw more robust conclusions regarding relational competence among healthcare professionals in mental healthcare settings. An overview of themes and codes is presented in Table 2. The results of the analysis are presented in detail in the following sections.

**Table 2:** Overview of themes and codes.

Themes	Codes	Relevant reseach/evidence
Having the ability to self-reflect and self-regulate	Self-reflection and awareness	Bhola et al., 2012; Brekke et al., 2018; Carmona-Navarro & Pichardo-Martinez, 2012; Gerace et al., 2018; Guansekara et al., 2017; Karman et al., 2015; Loughland et al. ,2015; Moltu & Binder, 2014; Tane et al., 2022; Vandervalle et al., 2020; Zugai et al., 2015
	Balancing own emotions	Basogul et al. 2018; Brekke et al., 2018; Gerace et al., 2018; Ljungberg et al., 2015b; Tane et al., 2022
Having a genuine interest in understanding the patient	Being attentive	Andvig & Biong, 2014; Brekke et al., 2018; Gerace et al., 2018; Guansekara et al., 2017; Moltu & Binder, 2014; Murphy et al., 2019; Vandervalle et al., 2020; Zugai et al., 2015
	Being interested in (and understanding) the unique patient	Brekke et al., 2018; Evatt & Scanlann , 2022; Gerace et al., 2018; Guansekara et al., 2017; Karman et al., 2015; Lamke et al. 2014; Ljungberg et al., 2015a; Ljungberg et al., 2015b?; Moltu & Binder, 2014; Moreno-Poyato et al.,2021; Murphy et al., 2019; Omylinska-Thurston et al., 2019; Romeu-Labayen et al. 2021; Vandervalle et al., 2020; Zugai et al., 2015; Zugai et al., 2017
	Being empathic	Basogul et al. 2018; Bhola et al., 2012; Gerace et al., 2018; Guansekara et al., 2017; Moreno-Payato & Rodriguez-Nogueira, 2020; Moreno-Poyato et al., 2021; Moreno-Poyato & Rodriguez-Nogueira, 2021; Murphy et al., 2019; Tane et al., 2022; Søvold & Solbakken, 2022
	Being sensitive to the person's needs	Andvig & Biong, 2014; Brown et al., 2014; Evatt & Scanlann , 2022; Gerace et al., 2018; Ljungberg et al., 2015a; Ljungberg et al., 2015b; Moltu & Binder, 2014; Moreno-Poyato et al., 2021; Omylinska-Thurston et al., 2019; Vandervalle et al., 2020; Zugai et al., 2015;
	Having a non-judgmental (accepting?) attitude	Brekke et al., 2018; Guansekara et al., 2017; Murphy et al., 2019; Romeu-Labayen et al. 2021; Tane et al., 2022; Vandervalle et al., 2020; Zugai et al., 2015; Zugai et al., 2017
	Having knowledge	Moltu & Binder, 2014; Ljungberg et al., 2015a ; Søvold & Solbakken, 2022; Zugai et al., 2017;
	Getting emotionally close enough	Moltu & Binder, 2014
Engaging in reciprocal interaction with the patient	Being approachable	Moreno-Poyato et al., 2021; Søvold & Solbakken, 2022; Vandervalle et al., 2020;
	Being available	Andvig & Biong, 2014; Brekke et al., 2018; Evatt & Scanlann, 2022; Gerace et al., 2018; Ljungberg et al., 2015a ; Moreno-Poyato et al., 2021; Romeu-Labayen et al. 2021; Tane et al., 2022; Vandervalle et al., 2020
	Being collaborative (equal)	Guansekara et al., 2017; Karman et al., 2015; Moltu & Binder, 2014; Søvold & Solbakken, 2022; Vandervalle et al., 2020; Zugai et al., 2015; Zugai et al., 2017;
	Having relational reciprocity (turn-taking)	Evatt & Scanlann, 2022; Guansekara et al., 2017; Li et al., 2019; Ljungberg et al., 2015a; Søvold & Solbakken, 2022
	Interacting with the patients	Brown et al., 2014; Evatt & Scanlann, 2022; Gerace et al., 2018; Lamke et al., 2014; Ljungberg et al. 2015a; Murphy et al., 2019; Vandervalle et al., 2020
	Sharing experiences and knowledge	Andvig & Biong, 2014; Brekke et al., 2018; Evatt & Scanlann, 2022; Guansekara et al., 2017; Ljungberg et al. 2015a; Søvold & Solbakken, 2022
	Being honest and open	Brekke et al., 2018; Gerace et al., 2018; Guansekara et al., 2017; Søvold & Solbakken, 2022; Vandervalle et al., 2020; Zugai et al., 2015;
	Sharing power	Ljungberg et al., 2015a
	Being reliable	Bhola et al., 2012; Ljungberg et al., 2015a
	Being personal involved	Ljungberg et al., 2015a; Vandervalle et al., 2020
Meeting the patient so that they feel acknowledged	Warmth and a sense of humor	Bhola et al., 2012; Brekke et al., 2018; Romeu-Labayen et al. 2021; Zugai et al., 2017
	Being respectful	Guansekara et al., 2017; Zugai et al., 2015; Zugai et al., 2017
	Being caring	Ljungberg et al., 2015a; Ljungberg et al., 2015b?; Zugai et al., 2015
	Being person-centered	Lamke et al., 2014; Ljungberg et al., 2015a; Tane et al., 2022; Vandervalle et al., 2020
	Being kind/friendly	Evatt & Scanlann, 2022; Ljungberg et al., 2015a ; Tane et al., 2022; Søvold & Solbakken, 2022
	Being flexible	Bhola et al., 2012; Evatt & Scanlann, 2022; Moltu & Binder, 2014; Zugai et al., 2017
	Being responding	Gerace et al., 2018;; Murphy et al., 2019; Moreno-Poyato & Rodriguez-Nogueira, 2021; Søvold & Solbakken, 2022
	Being steady/ Demonstrate commitment	Bhola et al., 2012; Brekke et al., 2018; Gerace et al., 2018; Vandervalle et al., 2020
	Being supportive	Andvig & Biong, 2014; Brekke et al., 2018; Hartley et al., 2022; Ljungberg et al.,2015a; Romeu-Labayen et al., 2021; Vandervalle et al., 2020; Zugai et al., 2015; Zugai et al., 2017; Søvold & Solbakken, 2022
	Decrease stressful situations	Lamke et al., 2014
	Being genuinely in communication	Battles & Berman, 2012
	Having courage	Brekke et al., 2018;
	Being patient	Bhola et al. (2012); Ljungberg et al., 2015a
	Creating hope	Andvig & Biong (2014), Brekke et al., 2018; Ljungberg et al., 2015b

## 3 Results

### 3.1 Overview of the literature

**Fig. 1:**
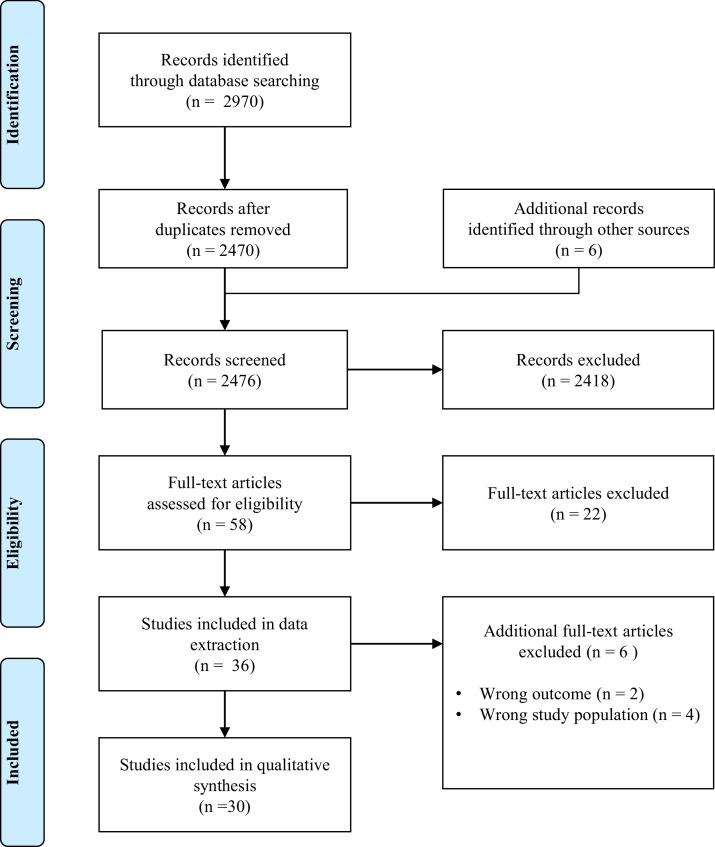
The flowchart of the article selection process.

The search results yielded a total of 2970 records (Fig. 1). After screening titles and abstracts, 58 articles were assessed in full text, of which 22 were excluded. The primary reasons for the exclusion of full-text articles were irrelevant outcome measures (*n* = 10), inappropriate study design (*n* = 4) and background articles (*n* = 3). The remaining 36 studies were included for data extraction. Six full-text articles were excluded due to irrelevant outcome measures (*n* = 2) and inappropriate study population (*n* = 4), namely studies focused exclusively on student populations or young people or with data from participants with and without mental health problems mixed in the same analysis.

In total, 30 scientific studies were included in this review, employing a variety of research methodologies to explore relational competence within mental healthcare. The studies were published between 2012 and 2023 and referred to research from in Australia (*n* = 6), USA (*n* = 5), Norway (*n* = 4), Spain (*n* = 4), UK (*n* = 4), Sweeden (*n* = 2), Belgium (*n* = 1), China (*n* = 1), India (*n* = 1), Netherlands (*n* = 1), and Turkey (*n* = 1). Ten studies were quantitative, 15 were qualitative, one was mixed-methods and four were reviews. The reviews contained a total of 120 articles, and the empirical studies involved in total 3010 participants and four cases. The participants were persons with lived experience, nurses, mental health nurses, milieu tehrapists, occupational therapists, psycho-therpists, undergraduated students, and junior medical officers. While all studies mentioned relational competence, it was neither extensively described nor precisely defined. Moreover, each study focused on different elements that could contribute to relational competence.

### 3.2 Themes

**Fig. 2:**
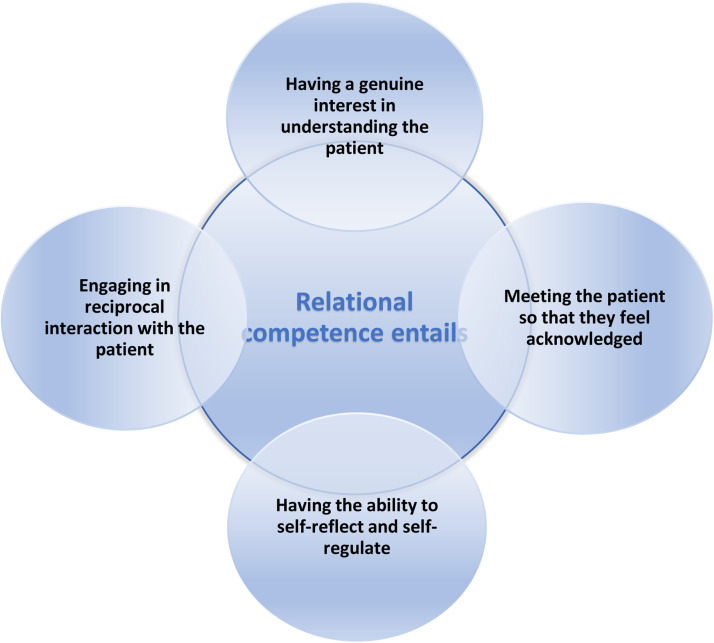
The four components of mental health professionals’ relational competence.

Four themes were found to describe relational competence in mental healthcare: *having the ability to self-reflect and self-regulate, having genuine interest in understanding the patient, engaging in reciprocal interaction with the patient, and meeting the patients so that they feel acknowledged* (Fig. 2). Each theme represents a central and vital aspect of relational competence, but fully developed relational competence must be understood as a whole in which all the themes are present.

#### 3.2.1 Having ability to self-reflect and self-regulate

Self-reflection entails being aware of one's own emotions and reflecting upon one's thoughts, and attitudes toward the patient. Moreover, it is necessary to separate one's own emotions from others’ (Gerace et al., 2018). Self-reflection allows the mental healthcare professional to be aware of their power and authority in a therapeutic relationship, a necessity when empowering the patient to target personal goals (Gunasekara et al., 2017).

Self-reflection is essential for self-regulation, meaning not acting upon feelings in an untherapeutic way in patient interactions. Patients experience mental health professionals acting in “autopilot mode” as non-helpful relationships (Ljungberg et al., 2016). A therapeutic relationship starts with the mental healthcare professional's self-awareness (Moltu and Binder, 2014), and without this competence it is difficult to maintain a therapeutic relationship with patients (Zugai et al., 2015). A negative relationship between stress in mental health professionals and emotional intelligence has been revealed. High levels of stress correspond with low levels of emotional intelligence and professional skills in the therapist (Sj et al., 2023). Mental healthcare professionals have experienced the negative impact of emotional distress and frustration in challenging situations in interaction with their patients. When coping therapeutically with such situations, it is necessary to be aware of one's own emotions and how to balance difficult emotions (Sj et al., 2023; Russell et al., 2022; Vandewalle et al., 2020). It is reported that patients find it difficult to deal with mental health professionals’ negative emotions (Ljungberg et al., 2016). There seems to be a correlation between nurses’ awareness of their own emotions and exposure to physical violence from patients (Başoğul et al., 2019). Nurses with higher degrees of emotional awareness were less exposed to physical violence, and those who better managed their own emotions were less likely to be subjected to psychological violence. This may indicate that emotional awareness may be a buffer against experiencing various types of violence in mental health settings (Başoğul et al., 2019). Moreover, nurses with greater emotional self-knowledge also showed a more positive attitude toward patients with suicidal behaviour and a better ability to handle the stress and anxiety that is often provoked in mental health professionals by this kind of behaviour (Carmona-Navarro and Pichardo-Martínez, 2012). Patients report being well cared for when their healthcare professionals are conscious of their role and do not burden the relationship with their own personal needs (Brekke et al., 2018).

Self-reflections and regulation may be strengthened in various ways. For example, mental health professionals’ self-reflection may benefit from patients’ feedback regarding their mental health professionals’ attitudes and behaviour (Karman et al., 2015). The ability to critically evaluate and question one's communication style seems to be important in professional practice. Improvement in this capacity was shown after attending a post-training communication programme (Loughland et al., 2015). Mental health professionals are found to profit from regular emotional processing or supervision where they can talk about challenges they face and self-reflect. Such support seems to be necessary for mental health professionals to become aware of feelings evoked by patients, which is essential for self-regulation, as well as to ensure their emotional well-being (Vandewalle et al., 2020; Tane et al., 2022).

#### 3.2.2 Having genuine interest in understanding the patient

Another theme revealed was a genuine interest in understanding the patient. This involves mental health professionals listening, questioning, providing choices and not being patronizing to their patients (Gerace et al., 2018; Brekke et al., 2018; Karman et al., 2015), as well as being sensitive to their patient's needs (Vandewalle et al., 2020; Brown et al., 2014; Evatt and Scanlan, 2022; Ljungberg et al., 2015; Moreno-Poyato et al., 2021). Being able to understand patients is essential for developing therapeutic relationships (Zugai et al., 2015; Tane et al., 2022), which build on professionals’ attachment to their patients, like thinking with their patients rather than of them (Karman et al., 2015). Being genuinely interested in understanding patients requires mental health professionals to be sensitive to patients’ needs in conversations where various themes of life are explored, encompassing existential and spiritual reflections (Andvig and Biong, 2014). To fully understand the individual patient's expression, mental health professionals also need theoretical knowledge of the nature of the patient's suffering and the disorder (Moltu and Binder, 2014; Ljungberg et al., 2015; Zugai et al., 2018). Theoretical knowledge is described to be necessary for being able to understand the patient (Gerace et al., 2018), however, patients emphasize that healthcare professionals must have both competence and experience, as well as engagement and commitment (Søvold and Solbakken, 2022).

Being understood by mental health professionals is highly valued by patients (Omylinska-Thurston et al., 2019), and in cases with good outcomes, mental health professionals can identify and understand their patients (Brekke et al., 2018; Murphy et al., 2019). Being empathic is important for patients (Tane et al., 2022; Søvold and Solbakken, 2022) and essential to understanding patients in mental healthcare (Gerace et al., 2018). An empathic mental health professional will try to take the patient's perspective, for example by asking the patient questions or employing introspective strategies using past experiences and switching places imaginatively with the patient to get insight into the patient's situation (Gerace et al., 2018). A combination of taking the patient`s perspective and empathic concern is associated with a stronger therapeutic relationship (Moreno-Poyato et al., 2021) and this combination seems to be especially important in the initial phase of the development of good connections with patients. In the later phase of the therapeutic relationship, or the working phase, empathic concern combined with reduced personal stress promotes collaboration around patient goal setting (Moreno-Poyato et al., 2021). Patients find it patronizing when mental health professionals are authoritarian or paternalistic (Ljungberg et al., 2016). Understanding the patient implies not only having empathy but also being a skilled communicator (Zugai et al., 2015), having good listening skills and showing the patient that one is present in the moment (Gerace et al., 2018), as well as a willingness to actively engage with others, fostering an environment where shared understanding can grow and expand (Gerace et al., 2018; Gunasekara et al., 2017).

Being genuinely interested in understanding patients may also require an accepting attitude. Mental health professionals should attend to patients’ narratives by presenting themselves as non-judgmental, being interested and willing to listen, and acknowledging the patient's emotions (Gunasekara et al., 2017; Vandewalle et al., 2020; Tane et al., 2022). Patients have reported the importance of confiding in their mental health professionals without being judged. Despite self-condemnation, patients experience respect and acknowledgment for being human when interacting with non-judgmental mental health professionals (Romeu-Labayen et al., 2022), and by experiencing acceptance they may become more self‐accepting (Murphy et al., 2019). A non-judgmental attitude creates a sense of safety and is essential for open communication and accessing patients’ thoughts and feelings (Bhola et al., 2012). Patients also report having the impression that their mental health professional is genuinely interested in understanding them when they experience their attention, authenticity, acceptance and compassionate spirit (Gunasekara et al., 2017; Zugai et al., 2015; Brekke et al., 2018). Mental health professionals must constantly show they care about their patients and that they are available for them (Evatt and Scanlan, 2022). When connected and attuned to their patients, mental health professionals will be more able to sense their needs and suffering (Vandewalle et al., 2020). Being attached and attuned to patients requires mental health professionals to actively use their physical sensations to cultivate a sense of closeness to understand their patients (Moltu and Binder, 2014).

#### 3.2.3 Engaging in reciprocal interactions with patients

Reciprocal interaction refers to sharing knowledge, responding to patients’ questions in understandable language (Ljungberg et al., 2015), and engaging in discussion of treatment options (Gunasekara et al., 2017), as well as sharing experiences from one's own life (Gunasekara et al., 2017; Brekke et al., 2018; Evatt and Scanlan, 2022; Ljungberg et al., 2015; Andvig and Biong, 2014). By demonstrating vulnerability and expressing their emotions through sharing, mental health professionals show humanness. This may have therapeutic benefits with positive recovery outcomes (Vandewalle et al., 2020) and promote more genuine relationships. It has also been found to be helpful when mental health professionals go beyond their expected professional role and offer something extra, for example, giving more time when needed (Ljungberg et al., 2015). However, it is important to know how to balance personal closeness and distance to maintain a professional therapeutic relationship (Bhola et al., 2012).

It is described as beneficial when mental health professionals are honest and open with their patients (Gerace et al., 2018; Gunasekara et al., 2017; Zugai et al., 2015; Vandewalle et al., 2020; Brekke et al., 2018; Tane et al., 2022; Søvold and Solbakken, 2022). Being transparent creates trust, which is essential for therapeutic relationships.

Reciprocal interaction also implies having a dialogue with the patient, exploring together (Evatt and Scanlan, 2022), and involving the patient in decision-making (Vandewalle et al., 2020; Ljungberg et al., 2015). Being genuine and transparent in contact with patients is described as helpful for therapeutic relationships (Gerace et al., 2018; Gunasekara et al., 2017; Vandewalle et al., 2020; Brekke et al., 2018). Reciprocal interaction may also involve engaging in everyday activities together and sharing experiences with patients (Vandewalle et al., 2020; Brown et al., 2014; Evatt and Scanlan, 2022). Being reliable and maintaining confidentiality are basic expectations in a reciprocal interaction (Ljungberg et al., 2015; Bhola et al., 2012).

To have reciprocal interactions with their patients, mental health professionals must be approachable (Vandewalle et al., 2020; Moreno-Poyato et al., 2021). Mental health professionals should demonstrate availability by spending time with their patients (Brekke et al., 2018; Tane et al., 2022; Evatt and Scanlan, 2022; Ljungberg et al., 2015), greeting them and inviting them to interact regularly and encouraging them and asking how to help (Gerace et al., 2018; Vandewalle et al., 2020; Andvig and Biong, 2014; Murphy et al., 2019). In terms of mental health professionals' communication style, patients value asking questions, listening, explaining and giving advice, as well as collaborating and sharing responsibility (Søvold and Solbakken, 2022). Being available and accessible and reaching out to patients is necessary for reciprocal interactions and establishing moments of togetherness (Vandewalle et al., 2020; Moreno-Poyato et al., 2021). Additionally, the timing of the therapist's responses is important; incorrect timing and lack of appropriate context of responses to patients have been shown to predict negative outcomes (Olson et al., 2023).

Patients value being seen as a fellow human being by mental health professionals (Gerace et al., 2018; Gunasekara et al., 2017; Ljungberg et al., 2015). They also value individual encounters with their healthcare professionals in a comfortable space without interruptions. Patients rate the therapeutic alliance better when therapists are experienced as flexible and able to alternate between dominance and submission in response to the patient's dominance-submission dynamic (Li et al., 2020). The contact should generate a bond of trust, which requires healthcare professionals to convey confidence, show warmth and offer patients the opportunity to share their thoughts and discuss any worries they may have (Moreno-Poyato et al., 2021), thereby demonstrating that they care for their patients (Romeu-Labayen et al., 2022).

Due to the power differential in the relationship, mental health professionals are responsible for balancing their power to achieve reciprocity (Zugai et al., 2015; Ljungberg et al., 2015). The use of power in a coercive manner is described by patients as traumatic (Ljungberg et al., 2016). There could be a tension between mental health professionals’ understanding of what a patient wants and their professional assessment of what he or she needs to mature. It is therefore necessary for the healthcare professional to have a cooperative mindset, considering the patient's needs for allowing growth based on the patient's premises (Moltu and Binder, 2014; Zugai et al., 2015; Karman et al., 2015; Brown et al., 2014).

#### 3.2.4 Meeting the patient so they feel acknowledged

Patients feel acknowledged when mental health professionals see them as people with unique qualities and perceptions and focus on their perspectives (Gerace et al., 2018; Gunasekara et al., 2017; Brekke et al., 2018; Ljungberg et al., 2015), as well as supporting them and their autonomy (Murphy et al., 2019). Mental healthcare professionals should care for their patients so they can experience being cared for (Zugai et al., 2015). Caring for patients entails remembering them, thinking about them and checking on them to make sure they are well (Ljungberg et al., 2015). If patients feel they are being ignored, they may find it harmful (Ljungberg et al., 2016).

Patients may be unstable, and various emotions can affect their perceptions of being acknowledged. A mental health professional with fully developed relational competence is able to respond to patients flexibly, dynamically adapting interactions to each unique patient and situation (Gerace et al., 2018; Evatt and Scanlan, 2022; Zugai et al., 2018; Murphy et al., 2019). When the therapist responds appropriately in each situation, the patient will likely feel genuinely acknowledged. This person-centred approach may be challenged in situations where the patient's needs and wishes are at odds with the treatment strategy. Mental health professionals must navigate such difficulties by balancing the patient's need for care, autonomy, professional knowledge and safety (Moltu and Binder, 2014; Zugai et al., 2018; Bhola et al., 2012). Being person-centred requires mental healthcare professionals to be connected and attuned to their patients (Vandewalle et al., 2020).

Being kind, and showing patients respect, compassion, trust, warmth and dignity, regardless of the situation, is essential to acknowledging patients in a therapeutic relationship (Gerace et al., 2018; Gunasekara et al., 2017; Zugai et al., 2015; Tane et al., 2022; Evatt and Scanlan, 2022; Ljungberg et al., 2015; Zugai et al., 2018). Complicated situations can be softened by using humour. Patients have cited humour as factor that facilitates therapeutic relationships as long as it conveys acceptance and is warm and non-authoritarian, bridging the distance between them and the mental health professional (Brekke et al., 2018; Romeu-Labayen et al., 2022; Bhola et al., 2012).

Patients appreciate mental health professionals who support them during both positive and challenging times, remaining by their side during relapses and episodes of mental distress (Zugai et al., 2015; Brekke et al., 2018; Ljungberg et al., 2015; Zugai et al., 2018). Being patient is seen as a personal strength (Ljungberg et al., 2015; Bhola et al., 2012). Facilitating encouraging conversations by expressing faith in possibilities is a response to patients’ difficulties that creates hope and acknowledges patients as individuals (Vandewalle et al., 2020; Brekke et al., 2018; Andvig and Biong, 2014; Murphy et al., 2019; Romeu-Labayen et al., 2022). If patients have acted in an unwanted manner, it is important for therapists not to react but rather respond in a non-judgmental way (Gerace et al., 2018). It is destructive if mental health professionals dwell on negative incidents (Ljungberg et al., 2016). Patients perceive mental health professionals as most empathic and feel acknowledged when there is congruence between verbal and non-verbal communication of acknowledgment and recognition (Battles and Berman, 2012). Additionally, it is important for patients that their relationships with mental health professionals be characterized by openness, trust and respect and that the quality of the communication itself is good (Søvold and Solbakken, 2022). This means that patients can ask questions, get answers, explore difficult topics and feelings, and express difficulties in the relationship. The characteristics of therapists that patients prefer in general are conscientiousness, kindness and emotional stability. When there is a high degree of congruence between these preferences and the actual characteristics of a therapist, the patient's satisfaction is higher. Furthermore, congruence between ideal and actual therapist characteristics seems to be predicted by the quality of the therapeutic alliance (Russell et al., 2022).

## 4 Discussion

The current analysis revealed that relational competence is a multifaceted phenomenon comprising four components: *having the ability to self-reflect and self-regulate, having a genuine interest in understanding the patient, engaging in reciprocal interaction with the patient, and meeting the patient so that they feel acknowledged.* While themes constructed may have significance separately, they are connected. The results do not suggest a hierarchy among them, which warrants further discussion.

As patients and situations may be inconsistent and changeable in the context of mental healthcare, mental health professionals must be highly skilled and capable of meeting each patient and situation with empathic support and safeguarding their autonomy and human rights (Beyene et al., 2019). Tailoring the relationship to each patient requires the integration of all four identified components of relational competence. This ensures professionals are equipped to navigate complex relationships with mentally ill patients whose emotions and behaviour are challenging. Each theme possesses unique meaning and relevance in different contexts, and when facing different patient challenges, yet they are interconnected in various ways. Our findings are very much in line with Travelbee's grand theory of human-to-human relationships (Travelbee, 1971) which emphasizes the importance of consciously using of one's own personality and knowledge, having the ability to take an interest in the patient as a person, being a fellow human being, and having a desire to help and to meet the patient's needs (Travelbee, 1971).

Central to maintaining a therapeutic relationship is the mental health professionals’ awareness of, reflection on, and regulation of their own feelings (Theme 1). For example, Gerace et al. (Gerace et al., 2018) showed that healthcare professionals’ ability to distinguish between their own and their patients’ feelings was crucial for maintaining therapeutic relationships. Similarly, Basogul et al. (Başoğul et al., 2019) emphasize that reflecting on one's feelings helps to reduce the risk of violent episodes from patients. Also, reflection and awareness may increase mental health professionals’ capacity to handle stress and anxiety associated with suicidal patients (Carmona-Navarro and Pichardo-Martínez, 2012). Our finding on the importance of mental health professionals’ self-reflection and self-regulation is in line with research on emotional intelligence that concludes that these qualities have a high impact on the quality of patient care (Raghubir, 2018). *Having the ability to self-reflect and self-regulate* is particularly important in situations where strong emotional reactions or conflicts unfold. Mental health professionals’ ability to understand and regulate their emotional reactions, thoughts, and behaviour can be decisive for maintaining a therapeutic approach and de-escalating during patients’ episodes of strong emotions and acting out behaviour.

Our findings show the importance of *having a genuine interest in understanding the patient* (Theme 2), a trait emphasized by patients and mental health professionals alike. Being empathetic and non-judgmental and listening helps to create trust and understanding in the therapeutic relationship. Empathy and unconditional acceptance were also highlighted by Rogers (Rogers, 1977) as particularly significant in terms of the therapist's demeanour. The content of the theme is based on descriptions from both qualitative (Gerace et al., 2018; Søvold and Solbakken, 2022) and quantitative studies (Zugai et al., 2015). The quantitative studies were also able to show that empathy, genuine interest, and understanding not only were important aspects of relational competence but also of particular importance in the establishment phase of a therapeutic relationship, as well as when the patient has a very complex background or extensive and multifaceted needs (Moreno-Poyato et al., 2021). In such settings, showing empathy, listening, understanding and considering the patient's perspective are critical parts of relational competence, which in turn is a prerequisite for developing a therapeutic alliance that promotes positive treatment outcomes. This is in line with prior research describing empathy, genuineness and warmth as important in various treatment approaches (Norcross and Wampold, 2011; Watson et al., 2014; Elliott et al., 2018). Professional knowledge is central and decisive for understanding the patient (Moltu and Binder, 2014; Ljungberg et al., 2015; Zugai et al., 2018) and is an important part of relational competence. However, professional competence must combine with empathy, engagement and commitment (Søvold and Solbakken, 2022). Considering that parts of relational competence are focused in all the included articles, one may wonder that knowledge of relational competence is not mentioned in any of them.

Key elements in being able to achieve reciprocal relationships (Theme 3) include healthcare professionals’ availability, initiative in interaction and time spent with patients (Gerace et al., 2018; Vandewalle et al., 2020; Brekke et al., 2018; Andvig and Biong, 2014; Murphy et al., 2019). Studies that shed light on the importance of *engaging in reciprocal interaction with the patient* in therapeutic settings indicate that open communication, being involved in decision-making processes and a safe atmosphere also are important for establishing a therapeutic alliance (Gunasekara et al., 2017; Vandewalle et al., 2020). This is consistent with research on therapeutic alliance and with the existing understanding of the alliance concept which, among other things, entails cooperation among patients and mental health professionals (Del Re et al., 2021).

*Meeting the patient so they feel acknowledged* (Theme 4) encompasses actions such as offering warmth, humour, flexibility, support and care or creating security in stressful situations. Several of the elements included in this theme are central aspects of emotional intelligence, as they require social awareness and the ability to interact according to the patient's needs and concerns (Raghubir, 2018). These elements are prerequisites for a good therapeutic alliance (Ackerman and Hilsenroth, 2003) and helpful relationships (Ljungberg et al., 2015) while also having an impact on the outcome of the treatment (Norcross and Wampold, 2011). Patients emphasize kindness, patience, supportiveness, and person-centred care as important for them to feel acknowledged (Ljungberg et al., 2015). Moreover, they highlight congruence, such as alignment between verbal and non-verbal communication or between desired and actual characteristics in the therapist, as the most important prerequisite for feeling acknowledged (Russell et al., 2022; Søvold and Solbakken, 2022; Battles and Berman, 2012). Congruence, which involves genuineness and authenticity in the therapist, is also one of the elements Rogers pointed out as crucial for the therapist's way of relating (Rogers, 1977).

There is no recipe for relational competence in a complex mental healthcare context. The nuances of patients’ mental states, the purpose of the relationship and the mental health professionals’ role in various situations are all factors that impact what is required of mental health professionals’ relational competence (McCormack and McCance, 2010). In navigating this complexity, mental health professionals encounter situations that demand a strong focus on acknowledging the patient, while other situations require a large degree of self-reflection and self-regulation. Diverse patient challenges, as well as the patient's relational competence, need to be considered alongside the dynamic nature of the therapeutic process, which emphasizes specific relational competence aspects at different treatment phases.

The interplay among the four identified components is illustrated by the reciprocal relationship revealed in our results. Genuine interest in understanding the patient in a respectful and non-judgmental way (Theme 2) not only fosters mutual interaction (Theme 3) but also enhances the patient's experience of being acknowledged (Theme 4). The interconnectedness of the themes highlights the holistic nature of relational competence and its impact on therapeutic outcomes.

While with some patients it can come quite naturally to have a genuine interest in understanding them, with others it can be very challenging. For instance, healthcare professionals often display negative attitudes toward patients with suicidal behaviour (Carmona-Navarro and Pichardo-Martínez, 2012), which impacts their ability to have a genuine *interest in understanding the patient.* Based on this we can assume that the *genuine interest in understanding the patient* is challenged in relationships with patients who display suicidal behaviour. Research shows that as many as one-third-of mental healthcare professionals experience physical violence and more than two-thirds of mental healthcare professionals experience psychological violence at work (Başoğul et al., 2019). Violent situations challenge mental healthcare professionals in balancing their emotions, linked to the theme *having the ability to self-reflect and self-regulate*.

Relational competence is dynamic and context-dependent. Even highly developed relational competence may be weaker on challenging days, influenced by factors like poor sleep, stress or pain. It is challenging for mental health professionals to consciously utilize the four elements of relational competence in a way that is best for the individual patient in various situations. Clinical supervision with reflections on interactions and relationships in clinical situations can strengthen practitioners’ understanding of self, others and relationships and can develop their relational competence (Beyene et al., 2019).

### 4.1 Strengths and limitations

This integrative review adopts a systematic and rigorous approach to synthesize a diverse array of literature to comprehensively understand the phenomenon of relational competence among healthcare professionals in mental healthcare settings. By adhering to Whittemore and Knafl's (Whittemore and Knafl, 2005) methodological framework for integrative reviews, this study ensures transparency and reliability in the review process.

The inclusive nature of the integrative review methodology is a major strength. Unlike traditional systematic reviews, this approach not only allows for the incorporation of studies with varying methodologies and evidence qualities but also specifically embraces both qualitative and quantitative research. This inclusivity broadens the understanding of relational competence among healthcare professionals in mental healthcare settings. In terms of methodologies, the studies included in our review employed various approaches such as qualitative, quantitative and mixed methods. The inclusion of literature reviews and a variety of research designs, including cross-sectional, action research and thematic analysis, contributes to a comprehensive understanding of relational competence. Furthermore, the methodological quality assessment conducted during the data evaluation stage enhances the credibility of the review findings, increasing confidence in the validity of the synthesized evidence.

Despite these strengths, several limitations must be considered. The review's reliance on studies published in English may introduce language bias, potentially excluding valuable perspectives from non-English sources and limiting diversity. The decision to exclude studies conducted prior to 2012, while justified, may have restricted the breadth of the review. It is essential to acknowledge that developments in the field may have occurred before this timeframe, and relevant insights from earlier studies might have been missed. Moreover, the thematic analysis, although robustly conducted using NVivo software, may introduce subjectivity in identifying and categorizing themes, potentially influencing the interpretation of findings. Moreover, the complexity and multifaceted nature of relational competence pose challenges in constructing a comprehensive search strategy. While efforts were made to address this, including using a research librarian and performing supplementary hand searches, it is possible that some relevant articles were not identified, thereby limiting the completeness of the review.

Also, it is worth noting that there are some limitations apparent in the data. For instance, there is a scarcity of studies from the patient perspective, and most of the included studies are focused on healthcare professionals such as nurses or psychotherapists. This skewness may impact the generalizability of the results to patient populations. Additionally, while the quality assessment indicates variations in study quality, no articles were excluded based on this assessment, underlining the commitment to inclusivity in the review process (Whittemore and Knafl, 2005). Some studies have been rated as high quality, while others have been assessed as having moderate or low quality. This suggests the importance of carefully evaluating the methodological strengths and weaknesses of each study and should be considered alongside the findings of this review.

## 6 Conclusion

The description of relational competence in this study encompasses four elements: *having the ability to self-reflect and self-regulate, having a genuine interest in understanding the patient, engaging in reciprocal interaction with the patient, and meeting the patient so that they feel acknowledged.* This highlights the multifaceted nature of relational competence in mental healthcare. It is important to recognize that these four themes are interconnected and interdependent. Relational competence in mental healthcare incorporates all these identified components. Each theme complements the others and contributes to building a strong therapeutic relationship between the patient and the mental health professional. To provide the best possible care for mentally ill patients, healthcare professionals must embrace and integrate these themes into their practice.

### 6.1 Implications for further research and practice

The findings of this study have significant implications for the development and enhancement of mental health care practices. Mental health professionals are unique individuals with different prerequisites for relational competence. For some, it comes naturally to acknowledge the individual patient, while for others it can be more challenging. Therefore, the development of relational competence is crucial for both students in higher mental health education and mental health professionals in clinical practice, equipping them with the necessary skills for effective professional relational work in mental healthcare. This requires considerable personal training and experience. Incorporating the identified themes into educational training programmes can empower healthcare professionals with the skills needed to navigate complex therapeutic relationships more effectively. Additionally, implementing feedback cultures or systems from patients in healthcare services can provide access to unique patient experiences and insights, which is crucial for developing and maintaining meaningful therapeutic relationships.

Furthermore, creating supportive environments and offering clinical supervision for healthcare professionals, as well as knowledge about relational competence, can promote self-regulation and reflection, emphasizing the importance of continuous professional development in evaluating and improving skills. These implications highlight directions for future research and interventions aimed at optimizing mental healthcare delivery and enhancing patient outcomes.

## Funding

This study is funded by the University of Stavanger and VID Specialized University. This research received no specific grant from funding agencies in the public, commercial, or not-for-profit sectors.

Lise Sæstad Beyene (LSB) is the main responsible for the study. All authors contributed to designing the study. The search string design was developed with the support of a qualified librarian, who performed test searches based on the terms and keywords elaborated. The search string was further developed in collaboration with LSB and HTS. The screening, inclusion of articles and quality check were carried out by all six researchers in pairs. The data extraction process was rigorous, with all authors (LSB, HEB, EBS, TLB, ARM and HTS) involved in the initial extraction process. Extracted data were systematically reviewed by LSB. LSB conducted the coding of the qualitative extracted data and systematized the codes into themes. The quantitative extracted data was coded and systematized into the existing themes by EBS. The themes were then validated by all authors. LSB, HTS and EBS have contributed to writing the manuscript, and all authors have contributed to revising the manuscript critically. All the listed authors are responsible for the content of the manuscript and have authority over the manuscript preparation as well as the decision to submit it for publication. The authors approved the submission of the current manuscript for International Journal of Nursing Studies Advances.

## CRediT authorship contribution statement

**Lise Sæstad Beyene:** Writing – review & editing, Writing – original draft, Visualization, Software, Project administration, Methodology, Investigation, Formal analysis, Data curation, Conceptualization. **Elin Bolle Strand:** Writing – review & editing, Writing – original draft, Validation, Methodology, Investigation, Formal analysis, Data curation, Conceptualization. **Aud Ragnhild Misund:** Writing – review & editing, Validation, Data curation, Conceptualization. **Helene Eidsmo Barder:** Writing – review & editing, Visualization, Validation, Data curation, Conceptualization. **Trine Lise Brente:** Writing – review & editing, Validation, Data curation, Conceptualization. **Hege Therese Størksen:** Writing – review & editing, Writing – original draft, Validation, Methodology, Data curation, Conceptualization.

## Declaration of competing interest

The authors declare that they have no known competing financial interests or personal relationships that could have appeared to influence the work reported in this paper.
